# A Novel Technique for Rapid-accurate 2D Hand Anthropometry

**Published:** 2017-06

**Authors:** Fereshteh AKBARNEJAD, Reza OSQUEIZADEH, Hamid Reza MOKHTARINIA, Amir Salar JAFARPISHEH

**Affiliations:** Dept. of Ergonomics, University of Social Welfare and Rehabilitation Sciences, Tehran, Iran

## Dear Editor-in-Chief

Anthropometry is one of the branches of ergonomics science to human body’s dimensions. Anthropometric measurement is classified into three different groups based on the used tools (1): manual anthropometry (Direct method), 2D anthropometry (photography method), and 3D anthropometry (1, 2). All of these methods have different pros and cons, however, in the direct method, researchers use tapes and calipers to measure the dimensions. The direct method has various disadvantages, such as inter and intra measure errors, the instrument position and orientation, pressure exerted by the measuring instrument, organ posture and identification of landmarks. Therefore, the operator should be skilled enough; however, this method is a time-consuming technique (1, 3, 4). Besides, if the mentioned errors are not controlled carefully, enormous data loss will occur (5).

In 1970s, an unconventional anthropometry method was developed to take pictures from human body. This method prevailed some of the direct method’s problems. The photography method enabled us to capture numerous pictures and store them safely for further processes (2). Nevertheless, the 2D method has its own bugs, such as perspective distortion, camera resolution, landmarking error and modeling error (6).

The 3D scanners have high accuracy, but the equipment and tools used for this method are expensive and also data transfer in this machine has some difficulties (3, 5). Furthermore, the machinery used in this method cannot be transferred easily (3). On the other hand, measurement using the 2D method and software is going to achieve much advancement. Therefore, the direct method is going to be replaced by the 2D technique. Since the 2D method has many advantages, such as being affordable and having high accuracy, so researchers prefer to use this technique (4). Nowadays, most of the anthropometrical surveys are carried out by means of 2D anthropometry (6). This method is used in various branches of medicine, such as monitoring facial shape, skin wound, teeth aberration, etc. (7).

Nowadays, MATLAB software is used for various numerical computations, mathematic algorithm analysis and image processing (8). Digital images are made from three major colors: red, green and blue, in which each pixel has its own color parameter (9). Since black and white images require less computational processes, they are more practical for most projects. Luminescence is one of the most common algorithms for producing black and white images (10). Edge detection and picture boundaries will be achievable by reducing unnecessary parts of the picture by means of the luminescence algorithm (3). In most researches canny algorithm has less edge fade (10).

This paper proposes a 2D hand anthropometry method that uses black and white pictures of the right hand using MATLAB software. The color images are converted to black and white by means of the luminescence algorithm, and then by means of canny edge detection algorithm, the important edges were detected. Afterward, using Sobel noise reduction algorithm image noise was reduced. Finally, the number of pixels in each of the 26 defined parameters are counted and converted to millimeters. In order to define the validity and reliability of the 2D technique and the traditional technique, 15 healthy volunteers (9 males and 6 females) with intact right hands participated in the study. In order to measure the linear dimensions a digital caliper, INSIZE 1108 series with the accuracy of 0.01 mm was used.

The Shapiro-Wilk test for normality distribution of each hand dimension was used. The results of test demonstrate that the data follow the normal distribution. Thereafter, correlation coefficients of Pearson method were used to evaluate the correlation of direct and 2D methods. Furthermore, in order to define the reliability of the 2D method, intra-class correlation coefficient (ICC) index was used. The results showed that the proposed 2D method meets the reliability requirements.

Based on the obtained results, it can be inferred that this method is a proper alternative for the direct anthropometry technique. Since the reliability and accuracy of this method is proven, it can be used for the anthropometric studies in other societies as well. Since the entire image, capturing process is automated, and also there is no human error during the measurements, reliability of the system is highly guaranteed.

## References

[1] Rogers MS Barr AB Kasemsontitum B Rempel DM (2008). A three-dimensional anthropometric solid model of the hand based on landmark measurements. Ergonomics, 51 (4): 511–26. 18357538 10.1080/00140130701710994

[2] Obi OF (2016). Hand anthropometry survey of rural farm workers in south-eastern Nigeria. Ergonomics, 59 (4): 603–11. 26207670 10.1080/00140139.2015.1073796

[3] Lin YL Wang MJ (2011). Automated body feature extraction from 2D images. Expert Syst Appl, 38 (3): 2585–91.

[4] Meunier P Yin S (2000). Performance of a 2D image-based anthropometric measurement and clothing sizing system. Appl Ergon, 31 (5): 445–51. 11059458 10.1016/s0003-6870(00)00023-5

[5] Al-amri SS Kalyankar NV Khamitkar SD (2010). Linear and non-linear contrast enhancement image. IJCSNS, 10 (2): 139–43.

[6] Habibi E Soury S Zadeh AH (2013). Precise evaluation of anthropometric 2D software processing of hand in comparison with direct method. J Med Signals Sens, 3 (4): 256–61. 24696802 PMC3967428

[7] Yu A Yick K Ng S Yip J (2013). 2D and 3D anatomical analyses of hand dimensions for custom-made gloves. Appl Ergon, 44 (3): 381–92. 23122430 10.1016/j.apergo.2012.10.001

[8] Kim S Casper R (2013). Applications of Convolution in Image Processing with MATLAB. University of Washington USA Available from: *https://pdfs.semanticscholar.org/391f/4dc0567f671b0718f80834fdc1e83a9fd54b.pdf?_ga=2.165313407.1773251993.1502694414-2035708052.1502694414*

[9] Kanan C Cottrell GW (2012). Color-to-grayscale: does the method matter in image recognition? PloS One, 7 (1): e29740. 22253768 10.1371/journal.pone.0029740 PMC3254613

[10] Maini R Aggarwal H (2009). Study and comparison of various image edge detection techniques. IJIP, 3(1):1–11.

